# Bisamidine Derivatives as Candidates for Tegumentary Leishmaniasis Therapy: Phenotypic Screening in Infection of Macrophages and Mechanistic Insights with Dual RNA-seq

**DOI:** 10.1021/acsomega.5c10727

**Published:** 2026-03-27

**Authors:** Joice Agripino, Ana C. Tahira, Luciana Ângelo de Souza, Matheus Silva e Bastos, Benjamin Sauer, Matthias Schmidt, Gustavo Costa Bressan, Raphael de Souza Vasconcellos, Wolfgang Sippl, Raymond Pierce, Murilo Sena Amaral, Sergio Verjovski-Almeida, Juliana Lopes Rangel Fietto

**Affiliations:** † Biochemistry and Molecular Biology Department, 28120 Universidade Federal de Viçosa, Viçosa 36570-900, Brazil; ‡ Laboratory of Cell Cycle, 196591 Instituto Butantan, São Paulo, SP 05503-900, Brazil; § General Biology Department, Universidade Federal de Viçosa, Viçosa 36570-900, Minas Gerais, Brazil; ∥ Institute of Pharmacy, 9176 Martin-Luther-University of Halle-Wittenberg, Halle (Saale) 06120, Germany; ⊥ Université de Lille, CNRS, Inserm, CHU Lille, Institut Pasteur de Lille, U1019 - UMR 8204 - CIIL - Centre d’Infection et d’Immunité de Lille, 59000 Lille, France; # Departamento de Bioquímica, Instituto de Química, 28133 Universidade de São Paulo, São Paulo, SP 05508-220, Brazil; ¶ http://a-paraddise.cebio.org/

## Abstract

*Leishmania braziliensis* is the primary causative agent of American tegumentary leishmaniasis (ATL), a critical parasitic tropical neglected disease. The chemotherapeutic arsenal has limited efficacy and significant toxic effects that lead to an urgent need to develop new medicines. Using the “drug repurposing” approach, histone-modifying enzyme inhibitors have been the subject of developing new drugs against neglected parasitic diseases. In this work, furamidine, a known diphenyl furan inhibitor of human Protein Arginine Methyltransferase (PRMT), along with a library of 31 developed analogues, was tested for leishmanicidal activity against *L. braziliensis* in the in vitro infection of macrophage assay. The most active and selective leishmanicidal analogue, BSF2 (EC_50_ of 0.64 μM (95% CI: 0.56–0.72), SI of 17.36), was further investigated by dual RNA-seq at 0.16 μM BSF2. The dual-transcriptome detected only 10 genes with significant differential expression (FDR ≤10%) in *L. braziliensis* related to ubiquitination, chromatin remodeling, and peroxisomal membrane transport pathways, following BSF2 treatment of infected macrophages. In addition, BSF2 had a significant effect (FDR ≤5%) on the expression of 577 genes in the infected macrophages, including the downregulation of TNF, IL-17, NF-κB, and Toll-like receptor pathways. This work opens new venues for developing new chemotherapy for leishmaniasis based on BSF2 or derivatives and highlights the dual transcriptome as a valuable phenotypic assay tool to investigate host–parasite interactions for antileishmanial drug discovery.

## Graphical abstract

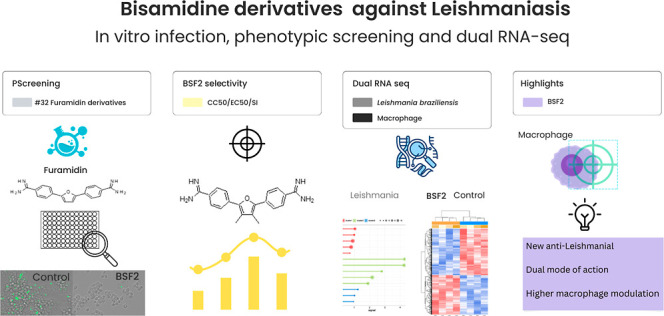

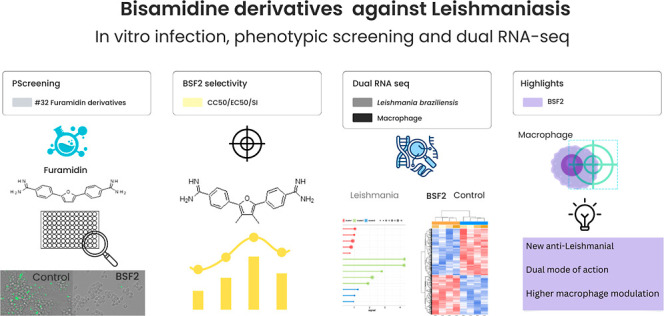

## Introduction

Leishmaniasis is considered to be a significant public health problem. According to the World Health Organization (WHO), 92 countries are considered endemic for cutaneous leishmaniasis (CL), with over 1 billion people living in endemic areas at risk of infection. Furthermore, it is estimated that 600,000 new cases of CL occur every year.^1^ In the New World, *Leishmania braziliensis* is the most prevalent parasite species that causes the American tegumentary leishmaniasis (ATL).^2–4^

ATL is a neglected disease with scarce treatment options that have questionable efficacy. However, chemotherapy is an essential aspect of control of the disease. The same compounds used decades ago are still in use, including pentavalent antimonials as the first-line treatment and pentamidine and amphotericin B as a second-line therapy for refractory cases. More recently, miltefosine and paromomycin have been used to treat ATL.^5–7^ Among the problems that chemotherapy for ATL presents are the severe side effects, high cost, low adherence to the treatment, and resistance acquired by the parasites.^8,9^ In addition, different susceptibilities to these drugs have been observed in clinical isolates of *L. braziliensis*.^10,11^ Therefore, searching for new therapies to treat ATL is increasingly necessary.

Trypanosomatids, such as *Leishmania* parasites, have a complex life cycle involving more than one host, different stages of cell differentiation, immune system evasion mechanisms, and survival in hostile environments. In addition, epigenetic gene regulation impacts parasite virulence, differentiation, and cell-cycle control.^12,13^ This epigenetic regulation occurs through mechanisms such as post-translational modifications (PTMs) of histones present in the chromatin nucleosomes of the cells.^14–16^ These PTMs are recognized by reader proteins that will recruit mechanisms to activate or repress gene transcription.^16–18^

Histone methylation is one of the best-characterized PTMs. Depending on the amino acid location of this modification on the histone, methylation results in the activation or repression of gene transcription. One such modification is the methylation of histone arginine residues, catalyzed by protein arginine methyltransferases (PRMTs).^16,19^ PRMTs control transcription of genes involved with several processes, such as DNA repair, cell signaling, and RNA metabolism.^16,19^ The balance between methylation and demethylation of histone proteins depends on the correct functioning of PRMT and demethylase enzymes (e.g., LSD1, JMJD6, and PAD4), and histone-modifying enzymes (HME) have been molecular targets for treating various diseases such as cancer and parasitic diseases.^16,20^

The five predicted PRMTs in *L. braziliensis* (LbPRMTs) have been functionally characterized by gene knockout.^21^ It is known that histone arginine methylation is present in all stages of the parasite life cycle in vitro, being higher in promastigotes than in amastigotes. None of the LbPRMTs proved essential for survival of the promastigote forms of the parasite.^21^ Still, the double deletion of PRMT1 and 5 or 7 affected the growth of amastigotes and compromised the infection of macrophages in vitro.^21^ LbPRMTs generally are predominantly cytoplasmic, except for LbPRMT6, which is more abundant in the nucleus. Furthermore, the interaction of each LbPRMT with a variable group of proteins, mainly RNA-binding proteins (RBPs), was revealed, indicating that the LbPRMTs act as epigenetic regulators of gene expression^21^ and reinforcing them as promising targets for antiparasitic therapies.

Using the “drug repurposing” approach, HME inhibitors of histone deacetylases (HDAC), PRMT, and LSD1 have been the subject of research aimed at developing new drugs against neglected parasitic diseases,^22,23^ including HDAC inhibitors against *L. braziliensis*.^24^ Efficacy of compounds analogous to furamidine, a diphenylfuran diamidine that has antimicrobial activity against parasites, has been reported against *Trypanosoma cruzi*, *Trypanosoma brucei*, *Plasmodium falciparum*, and *Leishmania donovani*.^25–29^ It is known that compounds containing diamidines have the ability to bind to genomic DNA in AT-rich local regions, thus inhibiting the binding of DNA binding-dependent enzymes and preventing gene transcription processes.^30^ In addition, diamidines have been identified by computational docking combined with biochemical assays to bind to and inhibit human PRMT1 and LSD1 histone-modifying enzymes.^28,31,32^

In this work, we screened a small library of 31 analogs of furamidine for leishmanicidal activity against *L. braziliensis* during macrophage infection. The compounds were evaluated for toxicity to macrophages as host cells, and those not toxic to macrophages were further evaluated for leishmanicidal activity. The best leishmanicidal compound, the furamidine analogue BSF2, was assessed for its effect on global gene transcription using dual RNA-seq analyses of macrophages infected by *L. braziliensis*. Our results show that this compound acts against parasite viability in an in vitro infection of macrophage assay, with a low EC_50_ (0.64 μM) and high selectivity index (17.36). While it exerts leishmanicidal activity, BSF2 has an immunomodulatory effect on *Leishmania*-infected macrophage gene transcription. Future experimentation is warranted to identify the mechanisms of action of BSF2, which might involve both binding to DNA at AT-rich sites or binding to the catalytic pocket of an HME. The data suggest the compound as a potential candidate to be further explored to treat ATL.

## Materials and Methods

### Parasite

*L. braziliensis* MHOM/BR/75/M2904-eGFP cell line that constitutively expresses the Green Fluorescent Protein^33^ was maintained in Grace’s Insect medium (Gibco, Life Technologies) supplemented with 10% heat-inactivated fetal bovine serum (LGC Biotecnologia, SP, Brazil), l-glutamine (2 mM) (Serva Electrophoresis and Life Science Products, NY, USA), and penicillin (100 μg/mL) (USB Corporation, OH, USA), pH 6.5 at 25 °C in a BOD chamber.

### Mammalian Cells

RAW 264.7 mouse macrophages (ATCC, Gaithersburg, MD, USA) were kept at 37 °C in a RPMI medium (RPMI-1640, Sigma-Aldrich, MO, USA) supplemented with 10% heat-inactivated fetal bovine serum (LGC Biotecnologia, SP, Brazil), l-glutamine (2 mM) (Serva Electrophoresis and Life Science Products, NY, USA), penicillin (100 μg/mL) (USB Corporation, OH, USA), and 25 mM HEPES (Fisher Scientific) from a 1 M stock solution, pH 7.2, in an atmosphere containing 5% CO_2_.

### Compounds

All tested bisamidine derivatives were synthesized and analytically characterized at the Institute of Pharmacy at the Martin-Luther-University of Halle-Wittenberg, Halle (Saale).^34^ All compounds were pure (>95%) and prepared as stock solutions at 10 mM in 100% DMSO. Furamidine and Amphotericin B were from Sigma-Aldrich (Merck KGaA, Darmstadt, Germany).

### Cytotoxicity to Mammalian Cells

Macrophages were cultured as mentioned above, and the protocol used to assess the toxicity of compounds by the resazurin assay was previously described.^33^ The screening test was done at 10 μM for each compound, and this concentration was based on the guidelines in hit and lead criteria in drug discovery for infectious diseases.^24^ For the cytotoxic concentration assay (CC50), the compounds were tested following the same protocol described in ref 35. Briefly, 96-well plates were assembled with 5 × 10^4^ macrophages per well and incubated for 24 h, as mentioned above. The cell density was adjusted based on counts performed in a Neubauer chamber, and cell viability was assessed by Trypan Blue exclusion. After 24 h, the metabolized medium was replaced with fresh medium, and the controls (Amphotericin B and DMSO) as well as the test compounds were added to the plates in quadruplicates. For CC_50_ determination, compound concentrations ranged from 80 to 1 μM. Plates were then incubated for 48 h and subsequently read on a microplate reader (SpectraMax M5) to assess resazurin metabolization as the indicator of cell viability at 570 and 600 nm.

### Macrophage Infection Assay

The macrophage infection assays and the determination of Effective Concentration (EC_50_) were performed using a previously described methodology based on *L. braziliensis*—M2904-GFP and following the protocol previously described that evidenced the ratio of 15:1 of parasite to macrophage as the best balance between infection efficiency and reproducibility, yielding optimal differences in fluorescence intensity between untreated control and compound-treated samples.^35^ Briefly, macrophages (1 × 10^5^ cells/well) were seeded in 96-well plates and incubated as described above. Infection was performed using stationary-phase promastigotes (seven-day culture) at a parasite-to-macrophage ratio of 15:1. Cell density was determined using a Neubauer chamber after dilution in 4% formalin, and parasite viability was evaluated indirectly prior to fixation by observing GFP-expressing promastigotes, indicating that they were alive and metabolically active. Efficient parasite internalization was confirmed by fluorescence microscopy, which showed a marked reduction in the number of extracellular promastigotes after 24 h of incubation. Wells were then washed with fresh RPMI to remove remaining extracellular parasites, and supplemental RPMI was added. Following an additional 24 h incubation, culture medium was replaced, and controls and test compounds (ranging from 80 to 1 μM for EC_50_ determination) were added in quadruplicate as mentioned above. Plates were incubated for 48 h, macrophages were lysed with RPMI containing SDS, and supplemented Grace’s medium was added. Plates were maintained at 25 °C in a BOD chamber for 4–6 days after macrophage lysis. This incubation period was required to allow for the differentiation of released amastigotes into promastigotes and their subsequent proliferation to reach the logarithmic growth phase, yielding a detectable fluorescence signal. Parasite survival was evaluated relative to that of untreated controls by measuring the fluorescence intensity of GFP-expressing parasites grown in the culture medium after lysis, using excitation at 490 nm and emission at 520 nm in a microplate reader (SpectraMax M5).

*Leishmania* in vitro infection varies with cell passage. To ensure the success of the in vitro infection, we used *L. braziliensis* with fewer than 14 passages. The number of passages of the macrophage cell line was not determined, but the same thawed batch of cells was used for all of the replicates, ensuring a consistent cell population across all replicates. The infection rates were measured as described below.

### Number of *Leishmania* per Infected Macrophage for Transcriptome Analyses

Initially, macrophages were infected at a ratio of 20 *Leishmania* parasites per macrophage following the same infection procedure described above. The higher *Leishmania*/macrophage ratio was chosen to ensure the presence of *Leishmania* mRNA sufficient for evaluation in the dual transcriptome, as it was expected to have lower parasite numbers after the treatments. Then, an independent determination of the average number of *Leishmania* per infected macrophage was obtained by fluorescence microscopy. For each of the four biological replicates in each group (DMSO control or BSF2-treated), the compounds were added at 1 EC_50_ (final concentration). DMSO was used at the same concentration as in the compound assays (0.1% v/v). Each sample was observed under 400× magnification (40× objective lens and a 10× eyepiece) on the microscope EVOS-FL (Life Technologies, Carlsbad, California, EUA) using GFP Light cube excitation light at 470/22 nm and observing emission at 525/50 nm. The number of infected macrophages and the number of parasites per macrophage were recorded. A total of 100 macrophages was analyzed per sample.

### Selectivity Index

The selectivity indexes (SIs) were calculated as the ratio CC_50_/EC_50_ obtained for RAW 264.7 macrophages (CC_50_) and *L. braziliensis* EC_50_ values.

### Statistical Analysis

Nonlinear regression analyses were performed in GraphPad Prism version 5.03. CC_50_ and EC_50_ values were obtained from concentration–response curves fitted with a four-parameter logistic model (4PL, Hill Slope model). Curves were generated using combined data from at least three independent experiments, each performed in quadruplicate. EC_50_ values were calculated from percent inhibition relative to untreated controls, and CC_50_ values were calculated from normalized macrophage viability data expressed as the percent of untreated controls using all technical replicates. Results are reported as best-fit values with 95% confidence intervals (95% CIs).

### RNA Sequencing Analysis in Response to Treatment with BSF2

The effect of BSF2 compound on the transcriptomes of macrophages and of *L. braziliensis* was evaluated by dual RNA-seq, using the macrophage infection assays described previously.^35^ Two conditions of infected macrophage cells were assayed, the BSF2-treated condition and the nontreated control condition (control containing DMSO at the same concentration used in the treated samples). Both conditions were assayed in *n* = 4 biological replicates each. Treatment was performed for 48 h at 0.16 μM BSF2, which is 1 of the EC_50_ established for BSF2 (0.64 μM). After treatment, the cells were removed from the bottle, collected, and cleared by centrifugation at 1200*g* at 4 °C for 10 min. According to the manufacturer’s manual, total RNA was extracted with the Trizol reagent (Qiagen) and frozen at −80 °C. Qubit and Nanodrop were used to assess the quantity and purity of total RNA. BioAnalyzer (Agilent) was used to assess the integrity of total RNA and to compute the RNA Integrity Number (RIN). Construction of cDNA libraries and RNA-seq was performed by GCB Sequencing and Genomic Technologies Shared Resource (Durham, NC). Sequencing was done in the Illumina platform using HiSeq 4000 with a read length of 150 bp paired-end. A total of 8 libraries were sequenced, representing the two conditions (BSF2-treated or control) with *n* = 4 biological replicates each.

Quality check of reads was performed using FastQC (v.0.11.9, https://www.bioinformatics.babraham.ac.uk/projects/fastqc/). Fastp (v0.20.0)^36^ was used to trim adapters and reads with low sequencing quality. To analyze the dual transcriptome, we built two different indexes using the *Mus musculus* genome (GRCm39:GRCm39.primary_assembly.genome.fa) with Gencode reference annotation (gencode.vM34.primary_assembly.annotation.gtf) available at Gencode page (https://www.gencodegenes.org/mouse) and *L. braziliensis* genome (TriTrypDB-68_LbraziliensisMHOMBR75M2904_2019_Genome.fasta) with reference transcriptome (TriTrypDB-68_LbraziliensisMHOMBR75M2904_2019.gff) available at TriTrypDb^37^ release 68. The sequenced reads were mapped using STAR (2.7.3a)^38^ against the mouse reference using Gencode adjusted parameters (--outFilterType BySJout, --outFilterMultimapNmax 20, --alignSJoverhangMin 8, --alignSJDBoverhangMin 1, --outFilterMismatchNoverReadLmax 0.04). To map against the *Leishmania* reference, the mapping parameters were modified to account for Splicing-leader sequences (SL) and to allow for more mismatches, once the *Leishmania* genome sequence is of lower quality compared with the mouse one. Thus, the alignments were performed using the same parameters as above except for two mapping parameters that were changed: outFilterMatchNmin 40 and --outFilterMismatchNoverReadLmax 0.15. For both STAR alignments, we used the parameter --outSAMattributes NH HI AS nM NM to allow for determining the best alignment of each read between the two reference genomes/transcriptomes. To choose the best alignment, we used the algorithm XenoFilteR (v1.6)^39^ in the R platform.^40^ Briefly, this algorithm compares the best alignment using an edit distance calculated by the MM threshold (Maximum number of edits threshold), and the read is assigned to the species with a lower edit-distance value. Here, we used the MM threshold of 8 and an unmapped penalty of 8. To split input reads between mouse and *Leishmania* source, the seqtk (1.2-r94) subseq tool^41^ was used and reads were remapped to their respective genomic index. The mapping quality check outputs were inspected with RSeQC (v3.0.1).^42^

Gene expression quantification was obtained using RSEM (v1.3.1);^43^ this algorithm can deal with multimapping reads through EM (expectation-maximization) calculation embedded in the algorithm. Differential gene expression analysis was performed on the macrophage RNaseq reads data set and separately on the *L. braziliensis* RNaseq reads data set, with the edgeR package,^44^ which calculates the gene expression changes in response to the treatment and determines whether the differences in expression levels among the experimental treated and control data sets are statistically significant. The counts of transcripts were used to perform differential analysis. Briefly, only genes that had CPM (counts per million) ≥1 in at least three replicates of one group (treated or control) were considered for further analysis. Preliminary analysis showed that the replicates were highly associated with transcription variance, then ComBat-seq^45^ was used to adjust counts using replicate as batch. To build the model, we used the following design: ∼group (mouse analysis) and ∼RIN + group (*Leishmania*). Only genes with at least FDR ≤10% (leishmania) and FDR ≤5% (mouse) were considered for further analyses. To maximize the discovery of biologically relevant signals in the *Leishmania* data set, which was characterized by low transcript abundance and high intragroup variability, we applied a more permissive False Discovery Rate (FDR) threshold of 10%. This adjustment was necessary to compensate for the reduced statistical power and attenuated effect sizes associated with low-count gene measurements, thereby facilitating a more comprehensive downstream functional analysis.

### *Leishmania* Enrichment Analysis

The enrichment analysis was performed using enrichment tools from TriTrypDb^37^ with GO and pathways analysis, using DEGs as input. To overcome the low number of DEGs, the STRING database^46^ was used. First, gene annotation was performed according to the match with the *Leishmania* protein sequence. Then, network properties were used to build a gene network using a maximum number of interactors of 20 in first and 10 in second shell and minimum required interaction score of 0.150, because the interaction network in the *Leishmania* protein database is scarce.

## Results

### Screening

In this work, we used the RAW 264.7 macrophage cell line and the previously produced *L. braziliensis* strain MHOM/BR/75/M2904-eGFP that constitutively expresses the green fluorescent protein GFP.^33^ This strain is capable of infecting the RAW 264.7 macrophage and the Balb/c mouse model, producing measurable cutaneous lesions.^35,47^

The synthesized analogs derived from furamidine were initially tested in the host cell model, RAW 264.7 macrophages, to assess their toxicity to these cells. Furamidine is a potent inhibitor of human PRMT1,^48^ has antiparasitic activity against parasites such as *T. brucei rhodesiense* ^49^ and *P. falciparum*,^50^ and served as the basis for the synthesis of the analogs tested in this work.

Amphotericin B was the positive control of the tests since it is used in leishmaniasis chemotherapy and has low toxicity to macrophages.^33^ Based on the 20% maximum toxicity of amphotericin B to macrophages, the nontoxic bisamidine derivatives were selected for further tests on the parasite in the infection assays. Thus, furamidine and 31 derivatives were tested, and only 5 showed high toxicity to macrophages, thus being eliminated: BSF6, BSF16, BSF20, BSF28, and BSF31 (Table 1). BSF6 was the most toxic compound to macrophages (55.85 ± 8.66 viable cells). The remaining compounds were tested for *Leishmania* infection. Eight compounds had a significant leishmanicidal effect (greater than 50%). BSF2 was the most effective against *L. braziliensis*, leading to 5.73% viability after treatment. BSF2 was even more active against *Leishmania* than furamidine, as seen in Table 1.

**Table 1:**
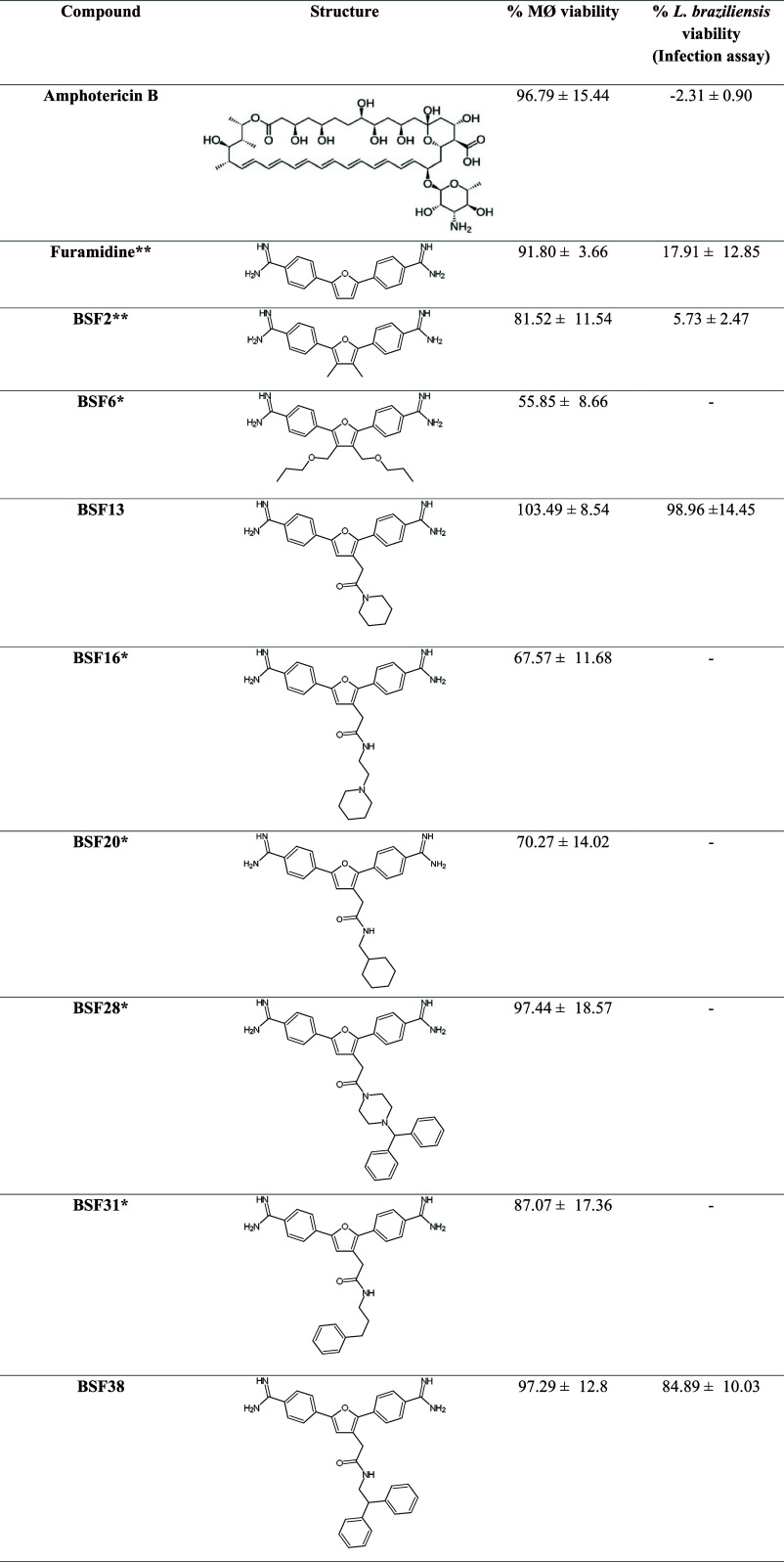

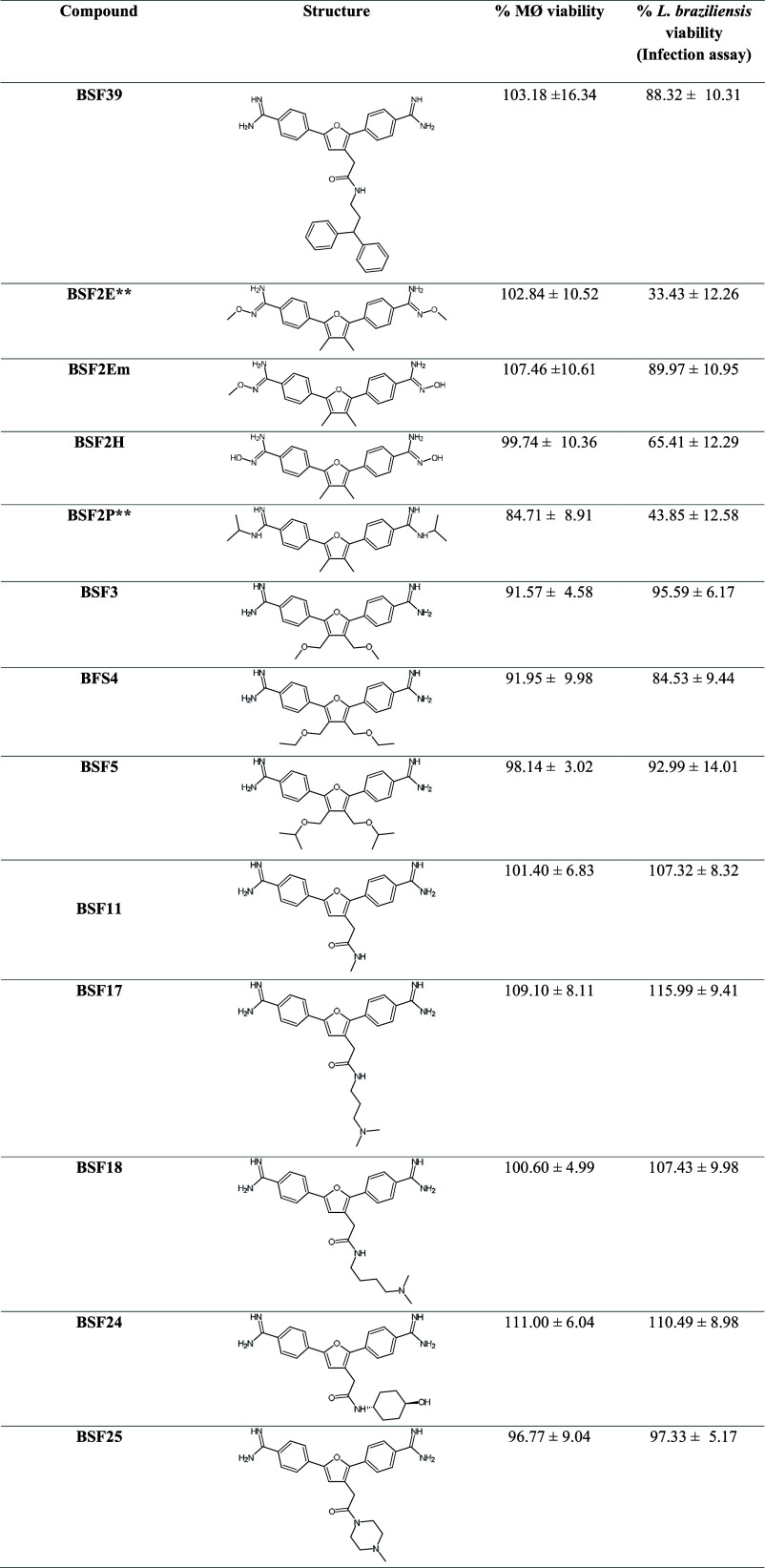

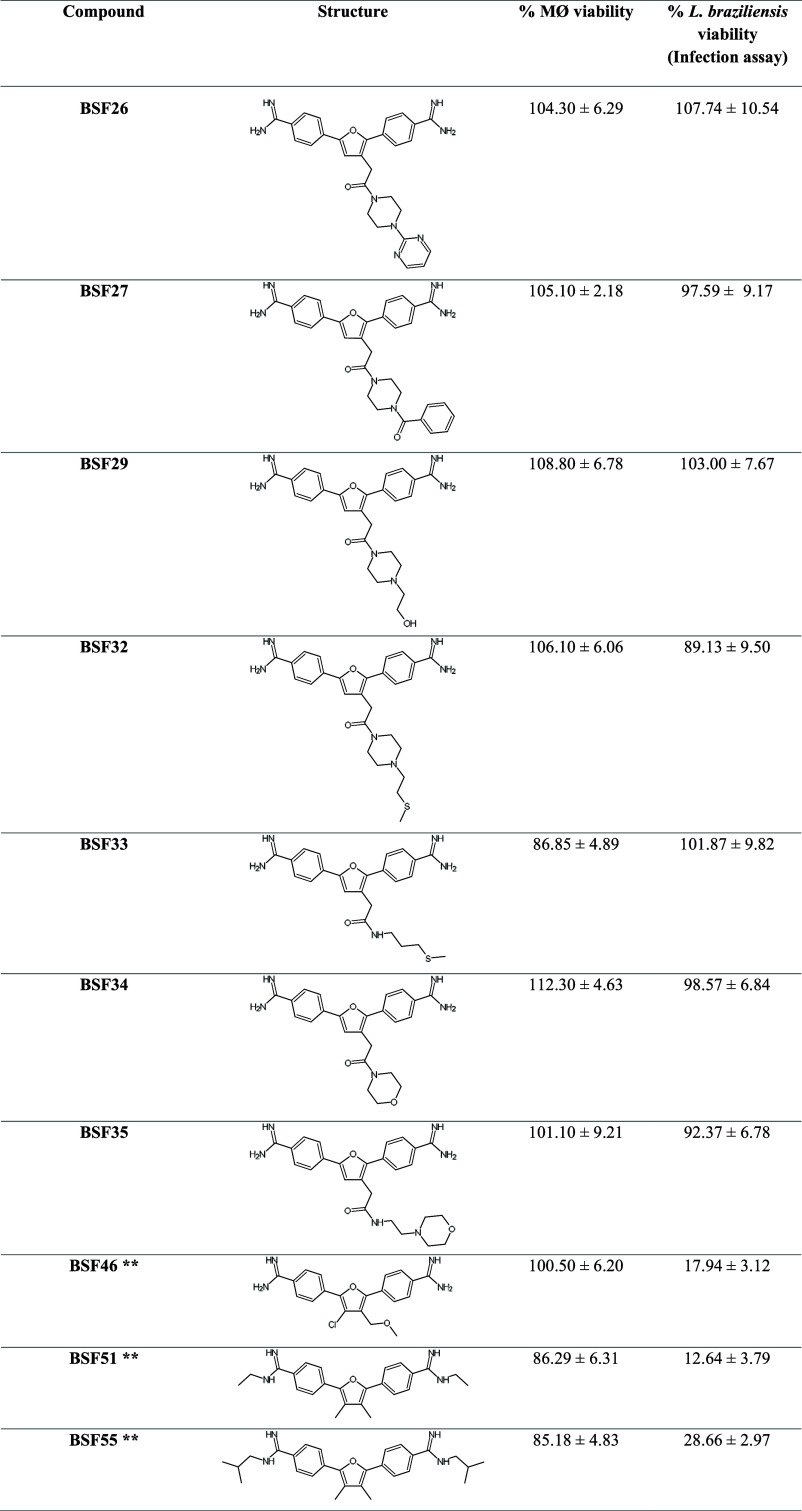
Screening Test of Bisamidine-Based Compounds: Evaluation of Toxicity to the Macrophage Host Cells and Effect on the Viability of *L. braziliensis* Intracellular Amastigotes

The data represent the median and standard deviation of at least three independent biological assays, each with technical quadruplicates. AmphB: amphotericin B. MØ: macrophages. *Compounds that were toxic to macrophages. **Compounds that were toxic to the parasites.

For the best eight furamidine derivatives, the CC_50_ to macrophages and EC_50_ to *Leishmania* viability in the infection assay were determined. This allowed the calculation of the selectivity index (SI), which reflects how much a particular compound is more selective for the parasite than for the host cell. Thus, as shown in Table 2, BSF2 was the most effective leishmanicidal compound against intracellular amastigotes of *L. braziliensis* (EC_50_ of 0.64 μM and SI of 17.36).

**Table 2:** CC_50_ and EC_50_ for the Selected Bisamidine Derivatives Acting on Macrophages and *L. braziliensis* Intracellular Amastigotes

compound	CC_50_ (μM) [95% CI]MØ RAW 264.7	EC_50_ (μM) [95% CI] *L. braziliensis*	selectivity index (SI)
BSF2	11.11 (9.00–13.70)	**0.64 (0.56–0.72)**	**17.36**
FURAMIDINE	8.48 (6.68–10.76)	11.84 (5.00–28.02)	0.72
BSF55	18.76 (12.23–28.76)	2.53 (0.55–11.60)	7.42
BSF2E	>80	14.31 (11.26–18.19)	>5.59
BSF2P	33.51 (25.06–44.83)	8.32 (4.55–15.23)	4.02
BSF46	11.80 (9.42–14.80)	NR	NA
BSF51	18.62 (12.24–28.32)	10.07 (4.00–25.29)	1.85

The data represent combined results from at least three independent biological assays, each performed with technical quadruplicates. Values are reported as best-fit estimates (μM) with 95% confidence intervals (95% CIs) in parentheses. MØ: macrophages. SI = CC50 (MØ)/EC_50_ (*L. braziliensis* amastigotes). NR: not reliably estimated (very wide 95% CI). NA: not applicable. SI was not calculated when EC_50_ was not reliably estimated. CC50 values reported as >80 indicate that 50% cytotoxicity was not reached at this highest concentration tested.

### Dual Transcriptome of Macrophages Infected by *L*.*braziliensis* and Treated with BSF2

Aiming to understand in a general way how the BSF2 compound acted against *Leishmania* during macrophage infection, we analyzed the transcriptome using dual RNA-seq cDNA libraries sequenced in the Illumina platform, corresponding to two experimental conditions of infected macrophages—treated and nontreated—in four biological replicates each. Treatment with BSF2 at 0.16 μM was performed, which corresponds to 1 of EC_50_ to allow for a residual infection to remain and thus permit the *Leishmania* transcriptome to be analyzed. The number of good-quality paired-end reads after trimming varied between 54,264,491 and 70,395,291 among the libraries. Most reads aligned to the mouse macrophage host cell genome with an average read mapping rate of 87% (54 million reads), while only an average of 2.89% (1.75 million reads) mapped to the genome of *L. braziliensis* *.* After assigning each read to its respective genome, the mapping rate was 85% (53 million reads) to the mouse genome and about 2.40% (1.5 million reads) to the genome of *L. braziliensis* (Figure S1 and Table S1). To evaluate if the number of reads mapped to the *Leishmania* genome corresponds to the experimental infection load that was independently determined, we performed a correlation analysis, and a high correlation value was observed (*r* = 0.80, *p* = 0.017, Figure S2), showing that the mapping rate was highly associated with the parasite load in macrophage cells. Following the quantification of reads per gene, we identified 24,285 (16.28%) genes expressed in mouse and 6521 (76%) transcripts expressed in *Leishmania*, showing that our sequencing and analyses pipeline did capture a large portion of the *Leishmania* transcriptome.

### Effect of BSF2 Treatment on the Global Dual Gene Expression

The principal component analysis (PCA) was plotted to analyze the global gene expression profile change on treatment with BSF2 and the degree of reproducibility of the replicate samples within each condition (Figure 1A). The two analyzed conditions were the nontreated *Leishmania*-infected macrophage control (DMSO control) and the BSF2-treated *Leishmania*-infected macrophages, after 48 h of treatment with BSF2 (0.16 μM).

**Figure 1:**
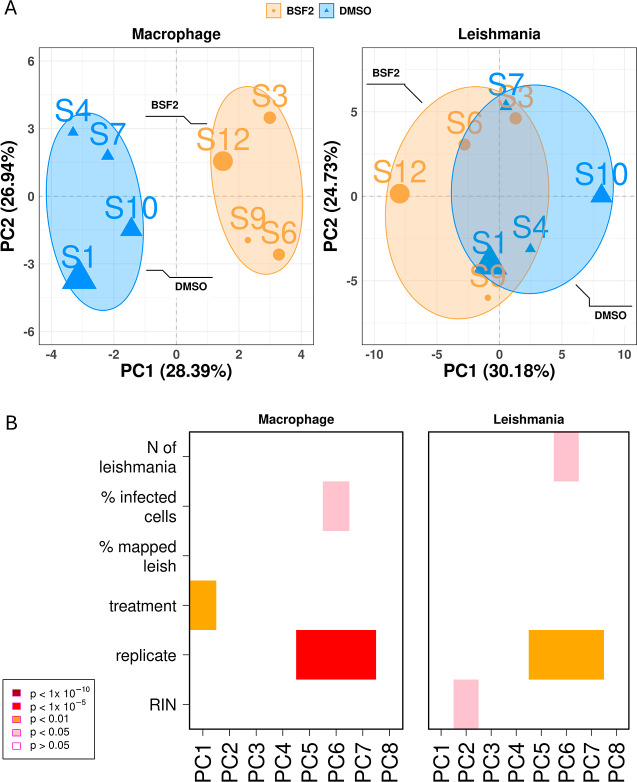
Global expression profile of macrophages (left panel) and of *L. braziliensis* (right panel) upon treatment with the BSF2 compound of macrophages infected with *L. braziliensis*. (A) Principal Component (PC) analysis of expression profiles. Colors and symbols indicate the experimental condition, namely, BSF2 treated (orange) or control (blue). The size of the symbols represents the relative load of intracellular forms of *L. braziliensis*, which was determined in a separate assay. The variance explained by each Principal Component (PC) is summarized in the axis title. (B) Association analysis of each PC with experimental variables. Each column represents a different PC, and each line shows an experimental assay variable. The significance of association is color scaled from dark red to pink, showing high and low significance of association, respectively, as indicated in the inset at left. RIN represents the RNA Integrity Number, which is a score that reflects the quality of the RNA (see Methods).

Treatment impacted the macrophage and the parasite differently, with the drug effect being much more pronounced in the macrophages (Figure 1A). In the macrophage RNA-seq data sets, the global gene expression profile was significantly affected by the treatment, as observed by PC1 values (PC1 ∼treatment, *p* <0.01, Figure 1B, left panel), while in the parasite data sets, the global gene expression profiles were only slightly influenced by treatment (Figure 1A), with the “treatment” variable not being statistically associated with any PC (*p* >0.05, Figure 1B, right panel); in the *Leishmania* transcriptome, the sources of variation were associated with the RIN quality score of the RNA, with replicates and with the *Leishmania* infection load of macrophages (Figure 1B, right panel). Unsupervised clustering of the *Leishmania* transcriptomic profiles revealed that two samples (S7 and S9) exhibited unconventional expression patterns, clustering with the opposing experimental groups rather than with their respective treatment group (Figure S3). However, biological replicates generally clustered according to treatment groups. This suggests a more complex and indirect effect on the parasite transcriptome.

### Treatment with BSF2 Modulates *Leishmania* Genes Involved with Chromatid Segregation, Ubiquitination, and Peroxisomal Complex

To understand the effect of BSF2 treatment, we focused on differentially expressed genes (DEGs). Comparisons among the *Leishmania* transcriptomes identified 10 DEGs with eight up- and two down-regulated genes under BSF2 treatment (FDR <10%, Table S2); the DEGs are involved in metabolism, chromosome organization, protein binding/ubiquitin binding, ubiquitin hydrolase, DNA binding, and ribosomal units.

To perform enrichment analysis, we used network properties in the STRING database,^46^ and only seven out of the ten DEGs were annotated in the database. This approach resulted in a network of 37 nodes and 140 edges, with an average node degree of 7.57 and an average local clustering coefficient of 0.595 (PPI enrichment p-value <1 × 10^–16^). The three DEGs not in the STRING database are LbrM.01.2.201150.1, LbrM.07.2.202220.1, and LbrM.28.2.208700.1, all annotated in the genome as pseudogenes, and likely do not affect the network analyses.

To explore the network, we used k-means clustering to build three groups, which can be observed clearly in Figure 2A. Cluster 1 (red) comprises 15 genes, of which three were DEGs (LbrM.34.2.210900.1, LbrM.29.2.002270.1, and LbrM.24.2.001970.1) upregulated under BSF2 treatment and are mainly associated with ubiquitination. Cluster 2 (green) with 14 genes is involved with peroxisomal membrane transport, highlighting gene LbrM.34.2.003170.1, which is highly connected and downregulated under BSF2 treatment. Cluster 3 (blue) is related to sister chromatid segregation and chromatin binding with two DEGs (LbrM.05.2.000410.1 and LbrM.19.2.001400.1) upregulated under BSF2 (Table S3). Gene LbrM.28.2.002610.1 (gray), annotated as Rft protein 2C, is not grouped in any cluster (Figure 2A). The pathway enrichment analysis and k-means clustering are summarized in Figure 2B and Table S3.

**Figure 2:**
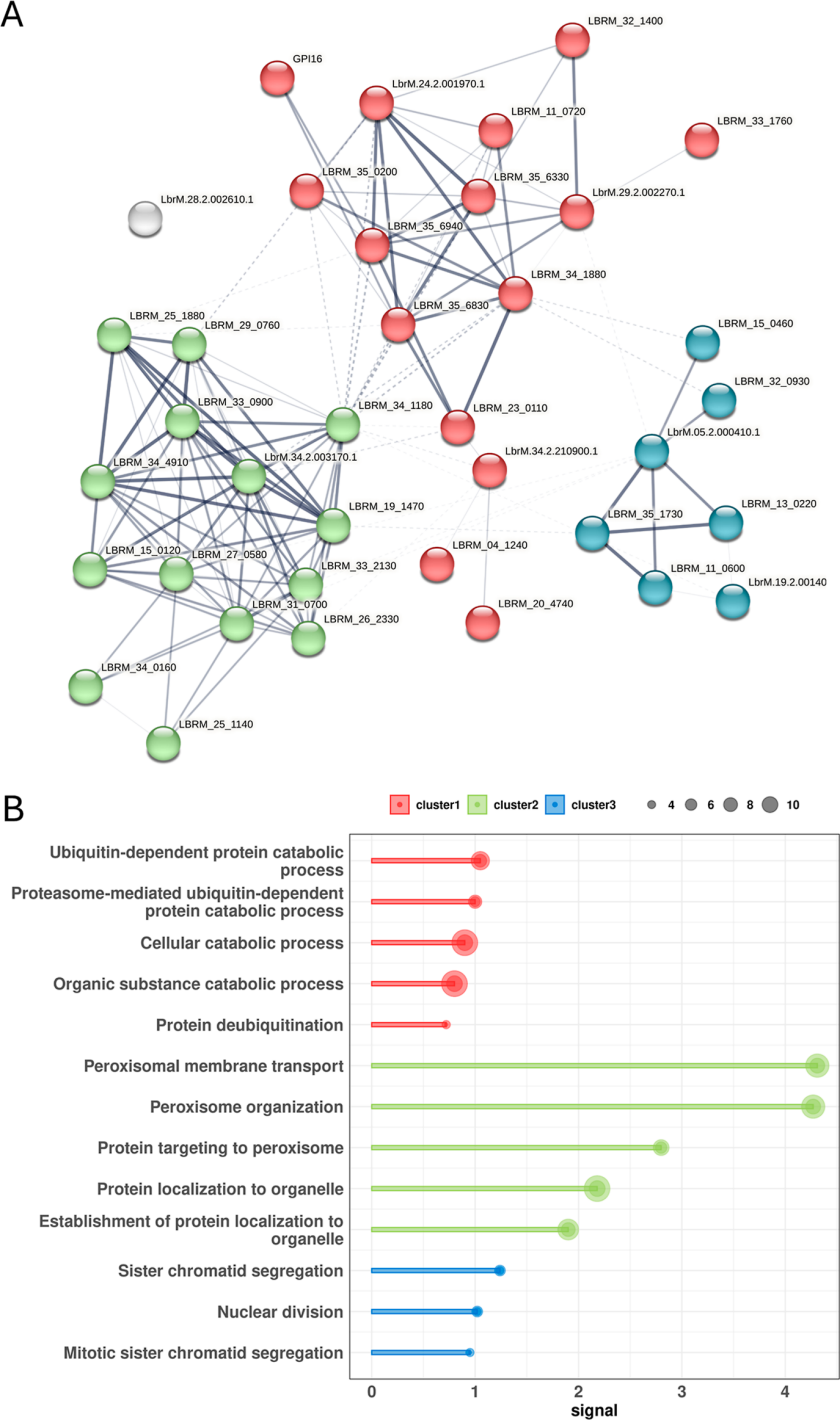
(A) Network analysis of DEGs in *Leishmania* treated with BSF2. Each node represents a gene/protein mapped in the STRING database. DEGs are labeled with “LbrM” lowercase “br” letters. Each color represents a cluster network: cluster 1 (red), cluster 2 (green), and cluster 3 (blue). Each edge is represented by a line, the thickness shows the confidence of the interaction. The dotted lines represent the interaction between clusters. (B) Pathway enrichment analysis. The pathways are represented in the *y*-axis and the signal strength is plotted at the *x*-axis. The size of the circle is proportional to the number of genes in the pathway, as indicated in the scale at the top. The colors correspond to each cluster subnetwork.

### BSF2 Treatment Downregulates Inflammatory Pathways in Mouse Macrophages

The effect of BSF2 on infection was quite noticeable among the macrophage transcripts. There were 577 transcripts differentially expressed (FDR ≤5%), of which 312 were downregulated and 265 upregulated (Figure 3A, Table S4). To investigate the pathways modulated after BSF2 treatment, we performed enrichment analysis with WebGestaltR.^51^ Using all up- and down-regulated DEGs, two gene ontology (GO) categories were identified as enriched, one of cellular component (CC) involved with a phagocytic cup (GO:0001891, seven genes, FDR = 3.31 × 10^–2^), and one of molecular function (MF) related to single-stranded RNA binding (GO:0003727, 13 genes, FDR = 3.66 × 10^–2^) (Figure 3B, Table S5). Using only downregulated genes, one gene ontology (GO), five KEGG pathways, and 26 REACTOME pathways were significantly overrepresented (FDR ≤5%) (Figure 3B, Table S5), while no pathway was found among the upregulated genes. Interestingly, pathways associated with inflammation (TNF, IL-17, and NF-κB signaling pathway) were composed mostly by downregulated genes. Inhibition of the TNF alpha signaling pathway caught our attention, as this cytokine has been associated with the response to *Leishmania* infection. Analyzing the downregulated genes related to TNFα, we identified genes encoding proteins present throughout the signaling pathway, such as Atf2, Atf4, Birc2, Cxcl2, Fos, Ikbkg, Junb, Mapk8, Mapk14, Table 2, Tnf, and Tnfaip3 (Figure 3C).

**Figure 3:**
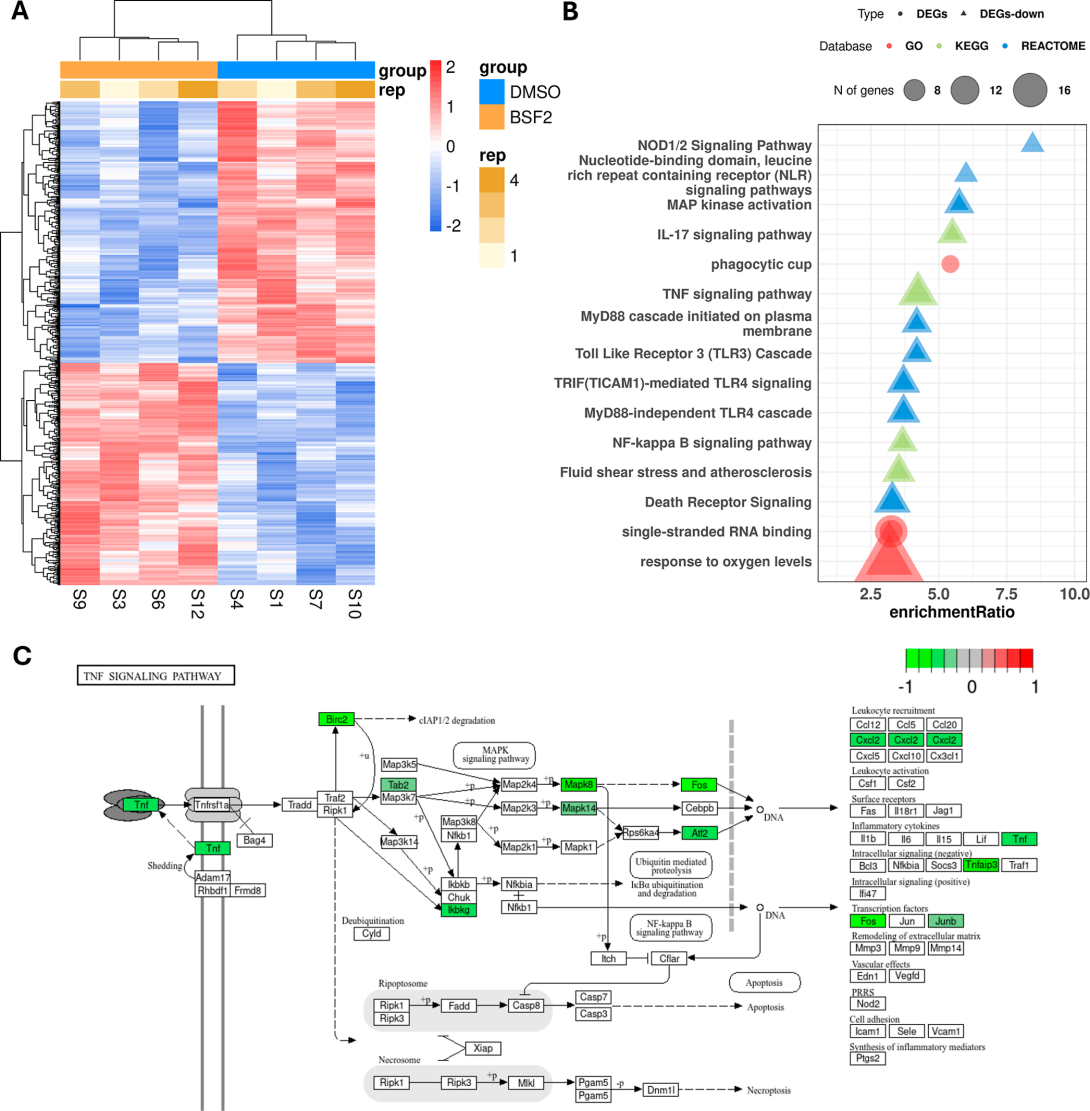
(A) Heatmap of 577 DEGs in macrophages. Each line shows a transcript and each column a sample. Gene expression levels are shown as z-scores (scale at right) in a color scale of blue to red, to represent low and high expression, respectively. Treated (BSF2) and control (DMSO) samples are marked in orange and blue, respectively. Each replicate batch is shown with a different brown color at the top of the columns. (B) GO term enrichment analysis of DEGs in infected macrophages treated with BSF2. Each line shows a category, or a pathway significantly enriched (FDR <5%). The *x*-axis shows the enrichment ratio (observed/expected gene counts). The enriched categories identified when using all 577 DEGs are represented in circles, while those identified when using only down-regulated DEGs are in triangles. The colors represent the database used in each analysis. The symbol size is proportional to the number of genes observed in each category/pathway. (C) Down-regulation of the TNF pathway from the KEGG database. Each gene/protein is shown in the rectangle with white background, while those marked with green are DEGs in our analysis, and the color scale at the top is proportional to log_2_(FC).

## Discussion

As highlighted in the literature, developing new drugs to treat leishmaniasis is urgent. In this work, we screened a small library of bisamidine derivatives. Furamidine is a known potent inhibitor of human PRMT1 ^48^ and has antiparasitic activity.^49^ Among the tested compounds, BSF2 was the most active leishmanicide against intracellular *L. braziliensis*, the principal causative agent of tegumentary forms of leishmaniasis in the New World; as observed in Table 2, BSF2 was a more effective leishmanicide than furamidine. Its EC_50_ is in the nM range (640 nM), and the selective index is higher than 10 (SI of 17.36). Indeed, this can be an interesting compound for developing a new therapy for ATL, since the guideline for drug discovery in infectious diseases of the developing world requires at least a 10-fold selectivity window for cytotoxicity using a mammalian cell line and an IC_50_ <10 μM against intracellular *Leishmania* ^24^ to classify a compound to go to the next step of development, which is the animal infection assay. Considering that hamster has been highlighted as a better model for mimicking the human infection by *L. braziliensis*,^52^ future in-depth investigation of BSF2 as a new chemotherapy candidate using hamster fresh cells and the animal infection is warranted.

Given the antiparasitic activity of BSF2 in vitro, we proceeded to investigate the potential mode of action by examining the transcriptional responses induced by the compound on the parasites and host cells. The dual transcriptome assay was performed at 1 of the BSF2 EC_50_ concentrations to ensure residual *L. braziliensis* infection, thus permitting the collection of live treated parasites and their transcriptome analysis. Although other higher sublethal doses were tested during assay optimization, 1 EC_50_ was selected, as it yielded sufficient parasites for RNA recovery. Under these conditions, it was apparent that the treatment exerted a more pronounced transcriptional effect on the macrophages than on the parasites.

In the *Leishmania* transcriptome, gene expression appeared to change as a function of RNA quality (RIN) and infection load, suggesting that the observed changes reflect indirect effects mediated by the altered intracellular environment due to treatment rather than a direct effect of BSF2 on the parasite. This suggests that host-derived alterations in the intracellular environment, resulting from the treatment, may be the primary drivers of changes in the transcriptional profile of the parasite under these conditions.

Despite the limited number of differentially expressed genes (DEGs) identified in the parasite transcriptome, network analysis revealed three enriched functional clusters that suggest stress-related responses. The interaction network is divided into three functional clusters: (1) ubiquitination (DEGs-up: LbrM.34.2.210900.1, LbrM.29.2.002270.1, and LbrM.24.2.001970.1); (2) sister chromatid segregation and chromatin binding (DEGs-up: LbrM.05.2.000410.1 and LbrM.19.2.001400.1); and (3) peroxisomal transport (LbrM.34.2.003170.1, DEG-down after the treatment).

Among the upregulated genes identified in *L. braziliensis* following BSF2 treatment, a prominent cluster is related to ubiquitination as well as to protein–protein and protein–nucleic acid interactions. Post-translational modifications (PTMs) play essential roles in regulating the life cycle of trypanosomatids, particularly during cellular differentiation triggered by infection.^13^ The enrichment of genes associated with ubiquitin signaling suggests that this pathway may be responsive to host-derived or BSF2-induced intracellular stress.

Ubiquitination is a versatile regulatory mechanism involved in cellular processes such as DNA repair, chromatin remodeling, protein trafficking, and cell cycle progression.^53^ This modification is reversible and can be tightly regulated by deubiquitinating enzymes (DUBs), which remove ubiquitin chains from substrate proteins, thereby adding another layer of control to this regulatory system.^54^ The upregulation of genes involved in ubiquitination in response to BSF2 suggests an adaptive stress response or a change in protease regulation within the parasite during intracellular infection.

Although still not completely understood, the ubiquitin–proteasome system in trypanosomatids has gained attention in recent years.^55^ Studies in *L. mexicana* have shown that DUB2, a nuclear deubiquitinase, is essential for the survival of promastigote forms as well as for the establishment and persistence of infection in mice.^56^ In our transcriptomic data, the gene LbrM.29.2.002270.1—annotated as a putative ubiquitin hydrolase and orthologous to *L. mexicana* DUB2—was upregulated following BSF2 treatment. This gene was coexpressed with others related to protein–protein and protein–nucleic acid interactions (LbrM.34.2.210900.1 and LbrM.24.2.001970.1), suggesting activation of cellular mechanisms involved in protein turnover and chromatin regulation. This expression pattern, even under sublethal drug concentrations, may reflect changes in the parasite that preserve protein homeostasis and chromatin dynamics in response to treatment-induced stress.

On the other hand, gene LbrM.34.2.003170.1, annotated as a member of the Pex19 protein family, was found to be downregulated following BSF2 exposure. Peroxins (Pex proteins) are essential for the biogenesis of peroxisomes and glycosomes.^57^ In *L. major* and *T. brucei*, Pex19 is indispensable for glycosome formation and mediates membrane protein import via interaction with Pex2 in *L. donovani*.^58^ Recently, the chemical inhibition of TbPex3— a Pex19 functional partner—was shown to disrupt glycosome integrity, resulting in parasite death.^59^ In this context, the downregulation of Pex19 in *L. braziliensis* suggests that glycosome biogenesis and metabolism may be compromised, contributing to the leishmanicidal effect of BSF2.

Treatment of infected macrophages with BSF2 resulted in a large transcriptional response of the host cells, reflecting significant modulation. Most of the differentially expressed transcripts showed reduced levels of expression. However, in the functional enrichment analysis, two gene ontology (GO) categories were upregulated: pathways associated with the phagocytic cup (GO:0001891) and RNA binding (GO:0003727), which suggests alterations in both phagocytic activity and post-transcriptional regulation of the host cell. This is expected, since activation of these mechanisms is observed in innate immune responses against pathogens: phagocytosis and rapid transcriptional control to direct the immune response.^60,61^

Among the negatively regulated pathways were those directly related to the inflammatory response, such as the TNF, IL-17, NF-κB, and Toll-like receptor (TLR) signaling pathways (Tables S4 and S5). Notably, BSF2 downregulated several genes in the TNF inflammatory pathway. TNFα production by macrophages infected with *L. braziliensis* is associated with parasite load reduction, but this also contributes to the development of severe skin and mucosal lesions.^62^ Reduction of the level of production of TNFα caused by BSF2 may have a protective effect on skin and mucosal lesions. In this line, macrophages infected by *L. donovani* also lose the ability to express c-fos and produce TNF in response to LPS stimulation.^63^

The TNF pathway is modulated by signaling from TLRs such as TLR2 and TLR4, whose expression is known to be increased in *L. braziliensis* infections, resulting in increased TNF production.^64^ In our study, TLR pathways (2, 4, and 9 known to recognize *Leishmania*) were suppressed after treatment with BSF2, contrary to what was observed in natural infections. Once again, it demonstrates the action of the compound interferes with immune response mechanisms.

Another gene related to the TNF pathway that showed decreased expression was Birc2/cIAP1 (cellular inhibitor of apoptosis 1). It encodes cIAP1, whose BIR domain binds TRAF2 in the TNF receptor (TNFR) complex, regulating stability and acting as an intermediary in TNFR signaling. In addition, cIAP1 can be recruited by complexes formed after TLR activation and promote the ubiquitination of target signaling molecules that activate NF-kB and MAP kinases.^65^ Downregulation of Birc2 promoted by BSF2 likely reinforces the suppression of such signaling.

The IL-17 pathway in BSF2-treated macrophages was significantly enriched in downregulated genes. IL-17 activates the expression of pro-inflammatory molecules related to leishmaniasis resistance,^66,67^ such as Cxcl2 (C-X-C motif chemokine ligand 2) and TNFα, which recruit other cells like neutrophils.^68^ Cxcl2 is overexpressed in macrophages infected with *L. braziliensis*,^69^ and its overexpression also depends on NF-kB, which is activated by IL-17, to stimulate TNFα production.^70^ The TNF pathway is also down-regulated following BSF2 treatment. Another gene with decreased expression in BSF2-treated macrophages is TNFAIP3 (TNFα-induced protein 3). TNFAIP3 acts as a negative downstream regulator of NF-kB- and TLR-mediated signaling, limiting pro-inflammatory responses.^71^

Our findings indicate that BSF2 may act not only through direct antiparasitic mechanisms but also by reprogramming the immune response of macrophages, possibly mimicking parasite strategies to suppress inflammation but, in this case, favoring the resolution of the infection. The immunosuppressive effect on the TNF, TLR, and pro-inflammatory transcription factor pathways may reflect a beneficial impact on containing the infection, without the effects associated with an exacerbated inflammatory response. Future studies could include other *Leishmania* species and macrophages of different origins to evaluate the effects of BSF2 treatment on the transcriptome. Combined therapy of a leishmanicidal immunosuppressant with other chemotherapies may improve the cure rate of CL and reduce healing time.^72^ Our results can open new avenues to future testing of BSF2 in combination with known leishmanicidal therapies. Nevertheless, we highlight that approaches beyond transcriptome analysis will be beneficial for a deeper understanding of BSF2 effects as a therapeutic drug, such as the evaluation of the influence of BSF2 on the *Leishmania* cell cycle, changes in the morphology of the intracellular parasite, and other approaches.^35^ Further elucidation of the mechanism of action of BSF2 will require future studies, including assays using antibodies against asymmetric and symmetric dimethylarginine (A/S DMA). In the present work, we focused on the impact of transcriptional control on expression to provide a general view of the possible roles of BSF2 in controlling infection. These data are useful to identify specific pathways for a deeper investigation in the future.

## Conclusions

This study conducted a thorough analysis of the potential of BSF2, a furamidine derivative, as a promising candidate for treating leishmaniasis, particularly tegumentary forms caused by *L. braziliensis*. Through a screening process, BSF2 exhibited notable leishmanicidal activity against intracellular *L. braziliensis*, outperforming furamidine. The BSF2 EC_50_ falls within the nanomolar range and has a high selective index, making it a compelling option for further therapeutic development. Dual transcriptome analysis was employed to investigate the effects of BSF2 on both the parasite and host cells. The transcriptome data indicated significant changes in gene expression in host cells, as an immunomodulator. Notably, BSF2 downregulated key pathways associated with innate immune response, including TNFα, IL-17, NF-kB, and Toll-like receptors. In the parasite transcriptome, the effect was less pronounced and related to expression modulation of genes involved with ubiquitination, chromatin remodeling, and peroxisomal membrane transport. This study underscores the potential of BSF2 as a therapeutic agent for leishmaniasis by targeting both the parasite and the host cell.

## Supplementary Material













## Data Availability

The raw RNA-seq data was deposited at NCBI under BioProject accession number PRJNA1334420.

## Notes

Mapped reads in genome index (XLSX)
Bar plot of mapped reads, plot of correlation between leishmania load and RNA-seq detection, and unsupervised clustering dendrogram of Leishmania replicates (PDF)
Differentially expressed genes in *Leishmania* samples (XLSX)
Enriched GO biological processes (FDR < 5%) of STRING network analysis (XLSX)
Differentially expressed genes in macrophage samples (XLSX)
Enriched categories and pathways of DEGs in macrophage samples (XLSX)

## ∇

Biochemistry and Molecular Biology Department, Universidade Federal de Viçosa, Brazil
