# Supplementary material for: Bisamidine Derivatives as Candidates for Tegumentary Leishmaniasis Therapy: Phenotypic Screening in Infection of Macrophages and Mechanistic Insights with Dual RNA-seq
Source: ACS Omega. 2026 Mar 27;11(13):20270–84. doi: 10.1021/acsomega.5c10727 (PMC13063000; doi:10.1021/acsomega.5c10727)
Supplement: Supplementary file 2 [file ao5c10727_si_002.pdf]

Supplementary File

Bisamidine derivatives as candidates for tegumentary leishmaniasis therapy: phenotypic screening in infection of macrophages and mechanistic insights with dual RNA-seq

Authors: Joice Agripino[1,7,*], Ana C. Tahira [2,*], Luciana Ângelo de Souza[3,7,a*], Matheus Silva e Bastos[1,7], Benjamin Sauer[4], Matthias Schmidt[4], Gustavo Costa Bressan[1,7], Raphael de Souza Vasconcellos[1], Wolfgang Sippl[4,7], Raymond Pierce[5,7], Murilo Sena Amaral[2,7], Sergio Verjovski-Almeida[2,6,7], Juliana Lopes Rangel Fietto[1,7].

Affiliations:

[1] Biochemistry and Molecular Biology Department, Universidade Federal de Viçosa, Brazil.

[2] Laboratory of Cell Cycle, Instituto Butantan, São Paulo, SP, Brazil.

[3] General Biology Department, Universidade Federal de Viçosa, Minas Gerais, Brazil.

[4] Institute of Pharmacy, Martin-Luther-University of Halle-Wittenberg, Halle (Saale), Germany.

[5] Université de Lille, CNRS, Inserm, CHU Lille, Institut Pasteur de Lille, U1019 - UMR 8204 - CIIL - Centre d'Infection et d'Immunité de Lille, 59000 Lille, France.

[6] Departamento de Bioquímica, Instituto de Química, Universidade de São Paulo, SP, Brazil.

[7] Consortium A-ParaDDisE – Anti-Parasite Drug Discovery in Epigenetics – http://a-paraddise.cebio.org

*Contributed equally to this work.

[a] Current address: Luciana Ângelo de Souza: Biochemistry and Molecular Biology Department, Universidade Federal de Viçosa, Brazil.

Corresponding author: Juliana Lopes Rangel Fietto, email: jufietto@ufv.br, Phone +55 31-36122464

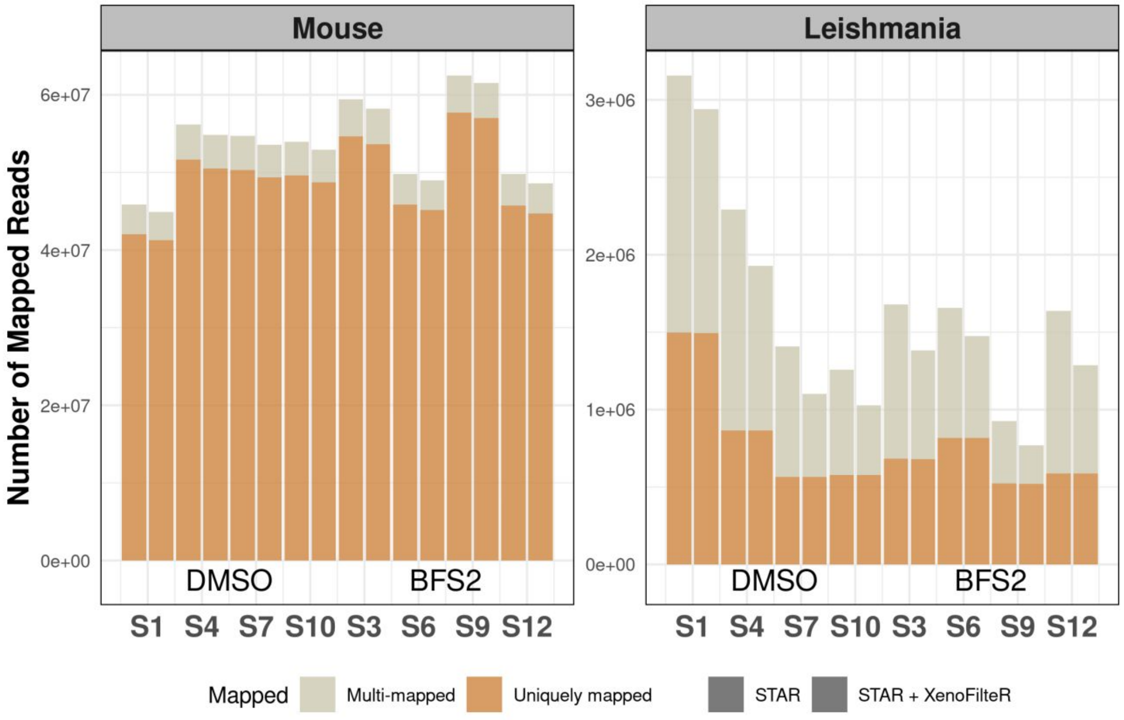

Figure S1: Barplot of mapped reads. Each bar represents the number of mapped reads in each sample using STAR (clean bar) or STAR + XenoFilteR (hashed bar). The multi-mapped and uniquely mapped reads are represented by colors grey and orange, respectively. Left panel shows reads mapped in mouse genome and right panel in Leishmania. The treated samples and non-treated samples are represented by BFS2 and DMSO labels under the bars.

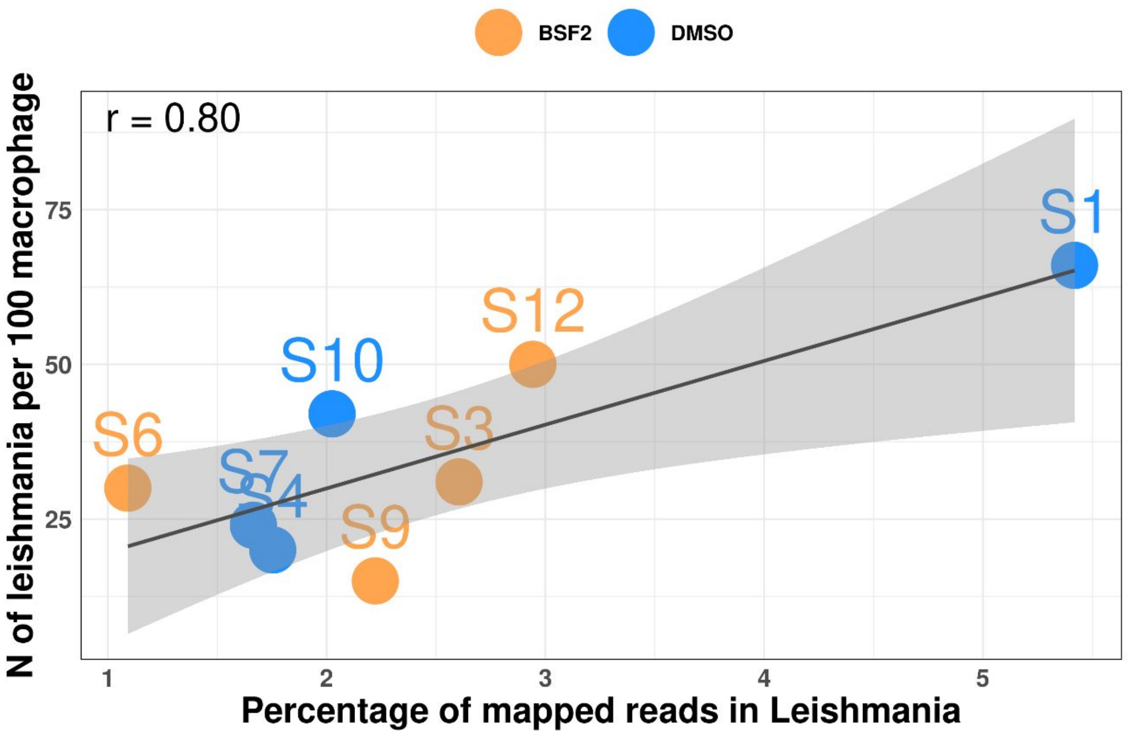

Figure S2: Plot of correlation between leishmania load and RNA-seq detection. The x axis shows the percentage of mapped reads in leishmania detected by dual RNA-seq analysis. The y axis shows the leishmania infection load measured in the assay, using the number of leishmania identified per 100 mouse macrophages. The samples are colored according to groups control (purple) and BSF2 treated (green). The linear model is marked by gray shadow and black line. The Pearson's correlation value is shown (r = 0.80).

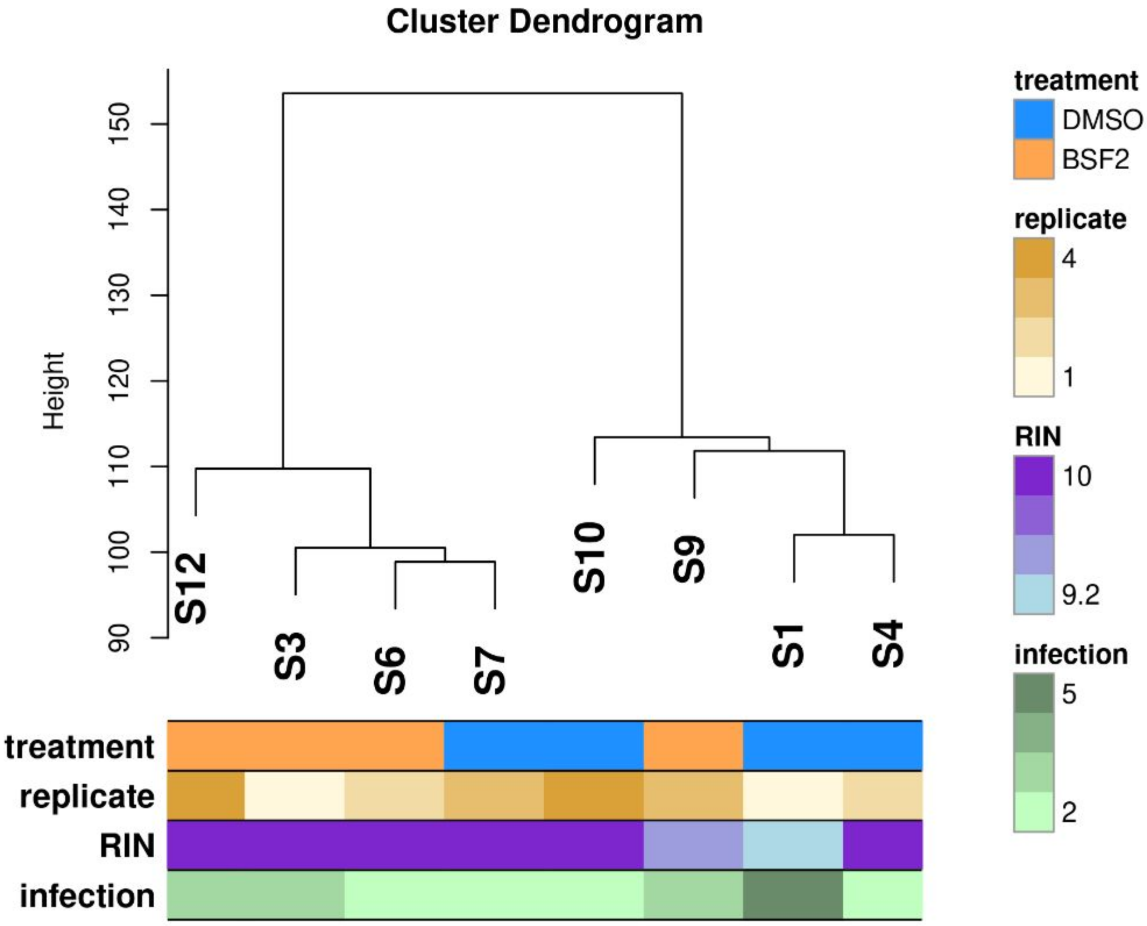

Figure S3: Unsupervised clustering dendrogram of Leishmania replicates. Dendrogram based on 6,521 expressed transcripts using Euclidean distance and the Ward.D2 clustering method. Each branch represents an individual biological replicate. Clearly, two groups were formed, one composed by S12, S3, S6 and S7 and the other by S10, S9, S1 and S4 samples. The heatmap below represents each variable value in the analysis. No metadata variables (RIN, infection load, or replicate) were significantly associated with these clusters. This result demonstrates that while the treatment is not the predominant driver of global transcriptomic variation, samples still exhibit a consistent tendency to cluster by treatment group.
